# The Association Between Quarantine Duration and Psychological Outcomes, Social Distancing, and Vaccination Intention During the Second Outbreak of COVID-19 in China

**DOI:** 10.3389/ijph.2022.1604096

**Published:** 2022-03-07

**Authors:** Lele Chen, Dingding Wang, Yuxin Xia, Renlai Zhou

**Affiliations:** ^1^ Department of Psychology, Nanjing University, Nanjing, China; ^2^ College of Education, Hebei Normal University, Shijiazhuang, China

**Keywords:** COVID-19, social distancing, psychological distress, wellbeing, vaccination intention, quarantine, China

## Abstract

**Objectives:** To examine the association between quarantine duration and psychological outcomes, social distancing, as well as vaccination intention during the second outbreak of COVID-19 in China.

**Methods:** A cross-sectional online survey was conducted in January 2021. Participants were invited to complete the measurement of quarantine duration, social distancing, psychological distress, wellbeing (WHO-5), and vaccination intention. Multiple linear regression and logistic regression were performed to examine the relationship between quarantine duration and psychological distress, wellbeing, social distancing, and vaccination intention.

**Results:** Of the 944 participants, 17.2% of the participants experienced quarantine. Quarantine for 1–7 days increased the social distancing (*β* = 2.61 95% confidence interval (CI) 1.90–3.33) and vaccination intention (OR = 2.16 95% CI 1.22–3.82). Quarantine for >7 days was associated with the increased social distancing (*β* = 3.00 95% CI 2.37–3.64) and psychological distress (*β* = 1.03 95% CI 0.22–1.86), and decreased wellbeing (*β* = 1.27 95% CI 0.29–2.26).

**Conclusion:** Longer quarantine duration showed increased social distancing, increased psychological distress, and decreased wellbeing. Quarantine for 1–7 days was associated with increased vaccination intention.

## Introduction

The Coronavirus disease (COVID-19) initially broke out in Wuhan, China, from December 2019 to March 2020 [1]. Although the epidemic abated in China, a second outbreak occurred in December 2020 in several provinces or cities in China such as Beijing, Hebei, and Liaoning [2, 3]. On 7 January 2021, a lockdown was firstly imposed in Shijiazhuang, the capital city of Hebei, to contain the spread of COVID-19 [4]. Mental health-related issues increased during the imposed quarantine, and this increase was referred as the second pandemic of mental health [5]. However, no empirical evidence is available on the impacts of quarantine on mental health during the second COVID-19 pandemic. In addition, as COVID-19 vaccines have been available from 31 December 2020 [6], whether quarantine affects vaccination intention is still not thoroughly studied. Thus, the exploration of the association between quarantine and vaccination intention as well as mental health during the second pandemic is necessary.

The evidence from a previous study has suggested that quarantine might be an effective way to contain the spread of COVID-19 in China and other affected countries [7], especially in the absence of a vaccine or effective treatment. However, quarantine has some considerable downsides. Previous studies have reported the direct consequences of quarantine including the increases of the social distancing, loneliness, and mental disorders [8–10]. A cross-sectional study performed in Wuhan showed that 21.7% of teenagers restricted inhomes were suffering from anxiety and 24.6% were suffering from depression [11]. Another cross-sectional study performed during the first outbreak in China showed that quarantine was associated with the increased risk of total psychological outcomes [12]; however, quarantine duration was not mentioned in this study. A recent review analyzed the psychological impact of quarantine and revealed that quarantined people reported negative psychological outcomes including stress symptoms, confusion, and anger [13]. Notably, only 3 papers out of 24 in this review explored the psychological impact of quarantine duration. Moreover, most of the papers in this review analyzed the evidence during the 2003 outbreak of severe acute respiratory syndrome and the 2014 Ebola outbreak [13]. Although the authors highlighted that quarantine duration may be responsible for the negative psychological repercussions of quarantine, the association between quarantine duration and psychological health need to be further studied in the context of the COVID-19 pandemic.

Increasing social distancing and vaccination may effectively hinder COVID-19 spread in public. When the second outbreak occurred in China, a decrease in social distancing was observed and was considered as quarantine fatigue [14], especially for those who experienced quarantine. A global survey showed that 90% of the participants in China had vaccination intentions when some vaccines were in clinical trials [15]. Whether this acceptance ratio can be maintained once the vaccines are available during the second outbreak is unclear. Moreover, several previous studies focused on some psychological or socioeconomic factors affecting vaccination intention instead of the effects of quarantine on the acceptance ratio of the COVID-19 vaccine [16–18]. Quarantined populations were more likely to worry about another quarantine or being infected than non-quarantined populations [19]. Estimating the relationship between quarantine duration and vaccination intention will help in promoting COVID-19 vaccination during the period of lockdown.

The present study is aimed to estimate psychological outcomes, social distancing, and vaccination intension during the second outbreak of COVID-19 in China. We analyzed the relationship between quarantine duration and social distancing, vaccination intention as well as psychological outcomes, namely psychological distress and wellbeing. This information is critical for preparing a protocol for future immunization programs against COVID-19 and preventing psychological disorders during the COVID-19 pandemic.

## Methods

### Participants

This online cross-sectional study was conducted during 10–23 January 2021, the period of the second outbreak of COVID-19 in China, when the people were under state-enforced strict lockdown. During this period, some cities such as Shijiazhuang in the Hebei province were locked down with traffic restrictions and the closure of work units. The details about the outbreak and the corresponding preventive measures have been described in past literature [20]. During the outbreak, individuals were invited to participate in the present web-based anonymous survey, which could be entered by scanning QR codes or clicking the linkage on Wechat. Self-reported questionnaires were administered to the participants through the Wenjuanxing platform (Changsha Haoxing Information Technology Co., Ltd., China). Finally, our electronic questionnaire was clicked 1,002 times. The inclusion criteria were being a Chinese citizen and being at least 18 years of age. We excluded participants aged <18 years and those who gave incomplete responses. Ultimately, the responses of 944 participants were analyzed in our study. The response rate was 94.2%. Among the included participants, 43.4% (*n* = 410) participants were from the Hebei province, 16.0% (*n* = 151) were from the Beijing city, and 18.4% (*n* = 174) were from the Liaoning province. No monetary compensation was provided to the participants. All participants provided their written informed consent for participation.

## Measures

### Quarantine Duration

Quarantine duration was measured by two questions: 1) “Did you experience quarantine before the second outbreak” and 2) “How long have you been quarantined.” As the public health center recommended, people were required to have quarantined for 7 days without exposure and 14 days with close contact. Thus, the quarantine duration was categorized into three groups: 0 days, 1–7 days, and >7 days.

### Psychological Distress

COVID-19 related psychological distress was measured as detailed elsewhere [21]. The following five items were inquired to reflect the participants’ psychological state during the COVID-19 pandemic: 1) I am nervous when I think about the current circumstances; 2) I am calm and relaxed; 3) I am worried about my health; 4) I am worried about the health of my family members; and 5) I feel stressed about leaving my house. The answers were coded on a 5-point scale, ranging from 1 (does not apply at all) to 5 (strongly applies), which indicated the extent to which each statement applied to the participant. The total scores of the psychological distress ranged from 5 to 25. The scale presented an acceptable Cronbach’s alpha score of 0.77.

### The State of Wellbeing

The World Health Organization-Five Wellbeing Index (WHO-5) was applied in the current study to measure the stated of wellbeing of the participants [22]. The WHO-5 recommends five positive items: “I have felt cheerful in good spirits”; “I have felt calm and relaxed”; “I have felt active and vigorous”; “I woke up feeling fresh and rested”; and “My daily life has been filled with things that interest me.” The extent to which the positive feelings were experienced in the last 2 weeks was scored on a 6-point Likert scale ranging from 0 (at no time) to 5 (all the time). The total score ranged from 0 to 25. The scale showed good internal consistency reliability of *α* = 0.91 in this study.

### Social Distancing

Increasing social distancing as another preventive intervention was encouraged for people when the city was under lockdown. Social distancing was determined during the second outbreak based on three questions concerning the coronavirus situation [21]. These questions mentioned the frequency of someone staying at home, not attending social gatherings, and keeping a distance of at least 2 m from other people for the past week based on a 5-point scale, ranging from 1 (Never) to 5 (Always). The total scores were summed in the current study. The total scores ranged from 3 to 15. The scale had an acceptable Cronbach’s alpha score of 0.77.

### Vaccination Intention

The COVID-19 vaccination intention was measured using a 1-item question: “The vaccine against COVID-19 infection has been available in the market, would you take it?” The answer was scored on a 5-point scale from “Definitely not” to “Definitely”. The participants who answered with “Definitely” or “Probably” were regarded as having an intention to take the vaccine. However, the participants who answered “Possibly,” “Probably not,” or “Definitely not” were regarded to have no intention to take the vaccine.

### Covariates

The covariates covered the demographic characteristics and health status. Demographic variables included gender (male or female), age (in years), residence (rural or urban), marriage status (married or unmarried), education (secondary and below or tertiary), and monthly per-capita income (≤1,000 yuan, 1,001–3,000 yuan, 3,001–5,000 yuan, or >5,000 yuan). The health status was measured by using a self-reported health status (fair/poor, good, or very good) and based on whether the participants had chronic diseases. The participants who had cardiovascular diseases, diabetes, hepatitis B, chronic obstructive pulmonary disease, chronic kidney diseases, or cancer were defined as having chronic diseases.

### Statistical Analyses

Frequency and mean were used to describe the characteristics of the study samples. Chi-square tests and *F-*test were used to compare the demographic differences among the participants with different quarantine duration. One-way analysis of variance (ANOVA) or Chi-square test was applied to explore the associations between quarantine duration and vaccination intention, social distancing, psychological distress as well as the stated of wellbeing. Logistic regression was applied to examine the association between quarantine duration and vaccination after adjusting for covariates. Odds ratio (OR) and 95% confidence intervals (95% CI) were computed. Multiple linear regression was performed to examine the association between quarantine duration and social distancing, psychological distress as well as the state of wellbeing after adjusting for covariates. To demonstrated the adjusted standardized difference of social distancing, psychological distress, and wellbeing among different quarantine durations, a forest plot was prepared based on multiple linear regression. The adjusted standardized score and 95% CI were calculated. Analyses were performed in the Stata version 15 (Stata Corp., College Station, TX, United States) and R version 3.4.3 (R Development Core Team, 2018). We considered a two-sided *p* value of <0.05 to be significant.

## Results

### Characteristics of the Study Sample

Of the 944 participants, 17.2% (*n* = 162) of the participants had been quarantined during the COVID-19 pandemic and 9.4% (*n* = 93) had been quarantined for at least 7 days. Table 1 shows that 72.9% of the participants were female 65.8% were based in urban areas, 53.8% were unmarried, and 89.0% of the participants had university degrees. The average age was 32.7 years. The health status of 28.7% of the participants was poor/fair and 8.8% had chronic diseases. In addition, the participants who had been quarantined were younger (*p* < 0.001) and unmarried (*p* = 0.001).

**TABLE 1:** Characteristics of the study population stratified by quarantine duration (Collected from 10 to 23 January 2021, China).

	Overall *N* (%)	Quarantine duration (days, *N*, %)			*p*-value^a^
		0 (*N* = 782)	1–7 (*N* = 69)	>7 (*N* = 93)	
Gender					0.150
Male	256 (27.1)	220 (85.9)	12 (4.7)	24 (9.4)	
Female	688 (72.9)	562 (81.7)	57 (8.3)	69 (10.0)	
Age, mean (SD), years	32.7 ± 13.0	33.4 ± 13.3	32.7 ± 12.3	27.3 ± 9.6	<0.001
Residence					0.099
Urban	621 (65.8)	511 (82.3)	53 (8.5)	57 (9.2)	
Rural	323 (34.2)	271 (83.9)	16 (5.0)	36 (11.1)	
Marriage					0.001
Unmarried	508 (53.8)	406 (79.9)	35 (6.9)	67 (13.2)	
Married	436 (46.2)	376 (86.2)	34 (7.8)	26 (6.0)	
Education					0.903
Secondary and below	104 (11.0)	87 (83.7)	8 (7.7)	9 (8.6)	
Tertiary	840 (89.0)	695 (82.7)	61 (7.3)	84 (10.0)	
Monthly per-capita income (Yuan)					<0.001
≤1,000	140 (14.8)	111 (79.3)	9 (6.4)	20 (14.3)	
1,001–3,000	286 (30.3)	224 (78.3)	20 (7.0)	42 (14.7)	
3,001–5,000	225 (23.8)	185 (82.2)	19 (8.4)	21 (9.3)	
>5,000	293 (31.1)	262 (89.4)	21 (7.2)	10 (3.4)	
Self-reported health					0.661
Fair/poor	271 (28.7)	231 (85.2)	18 (6.6)	22 (8.2)	
Good	482 (51.1)	396 (82.2)	34 (7.1)	52 (10.8)	
Very good	191 (20.2)	155 (81.2)	17 (8.9)	19 (9.9)	
Chronic diseases					0.155
No	861 (91.2)	707 (82.1)	65 (7.5)	89 (10.4)	
Yes	83 (8.8)	75 (90.4)	4 (4.8)	4 (4.8)	

Note:

a F-test or χ2 tests as appropriate.

### Univariate Analyses of the Association Between Quarantine Duration and Psychological Outcomes, Social Distancing, and Vaccination Intention

Table 2 shows the results of the univariate analyses of the association between quarantine duration and psychological distress, wellbeing, social distancing as well as vaccination intention. The results of one-way ANOVA analyses showed that quarantine duration affected psychological distress (*p* = 0.003), wellbeing (*p* = 0.016), and social distancing (*p* < 0.001). The least significant difference test results showed that participants who had been quarantined for >7 days had the largest psychological distress (17.2 ± 3.8) and social distancing (13.3 ± 1.9) as well as the poorest wellbeing (15.2 ± 5.4) compared with those without quarantine. Overall, 62.1% of the participants intended to take a COVID-19 vaccine, whereas 37.9% did not intend to take the vaccine. The chi-square test results showed that participants who had been quarantined during the pandemic had a higher vaccination intention than those who had not been quarantined (*p* = 0.005).

**TABLE 2:** Univariate analyses of the association between quarantine duration and psychological distress, wellbeing social distancing as well as vaccination intention (Collected from 10 to 23 January 2021, China).

	Overall	Quarantine duration (days)			*p*-value^a^
		0 (*N* = 782)	1–7 (*N* = 69)	>7 (*N* = 93)	
Psychological distress	16.0 ± 3.9	15.8 ± 3.9	16.4 ± 3.9	17.2 ± 3.8^b^	0.003
Wellbeing	16.5 ± 4.8	16.6 ± 4.7	16.1 ± 4.6	15.2 ± 5.4^b^	0.016
Social distancing	10.7 ± 3.1	10.1 ± 3.1	12.7 ± 2.6^b^	13.3 ± 1.9^b^	<0.001
Vaccination intention					0.005
No	358 (37.9)	315 (40.3)	18 (26.1)	25 (26.9)	
Yes	586 (62.1)	467 (59.7)	51 (73.9)	68 (73.1)	

Note:

a F-test or χ2 tests as appropriate.

b The comparison is declared significant when using the least significant difference (LSD) test and selecting the participants without isolation as reference group.

### Multivariate Analyses of the Association Between Quarantine Duration, Social Distancing, and Psychological Outcomes and Vaccination Intention

**FIGURE 1:**
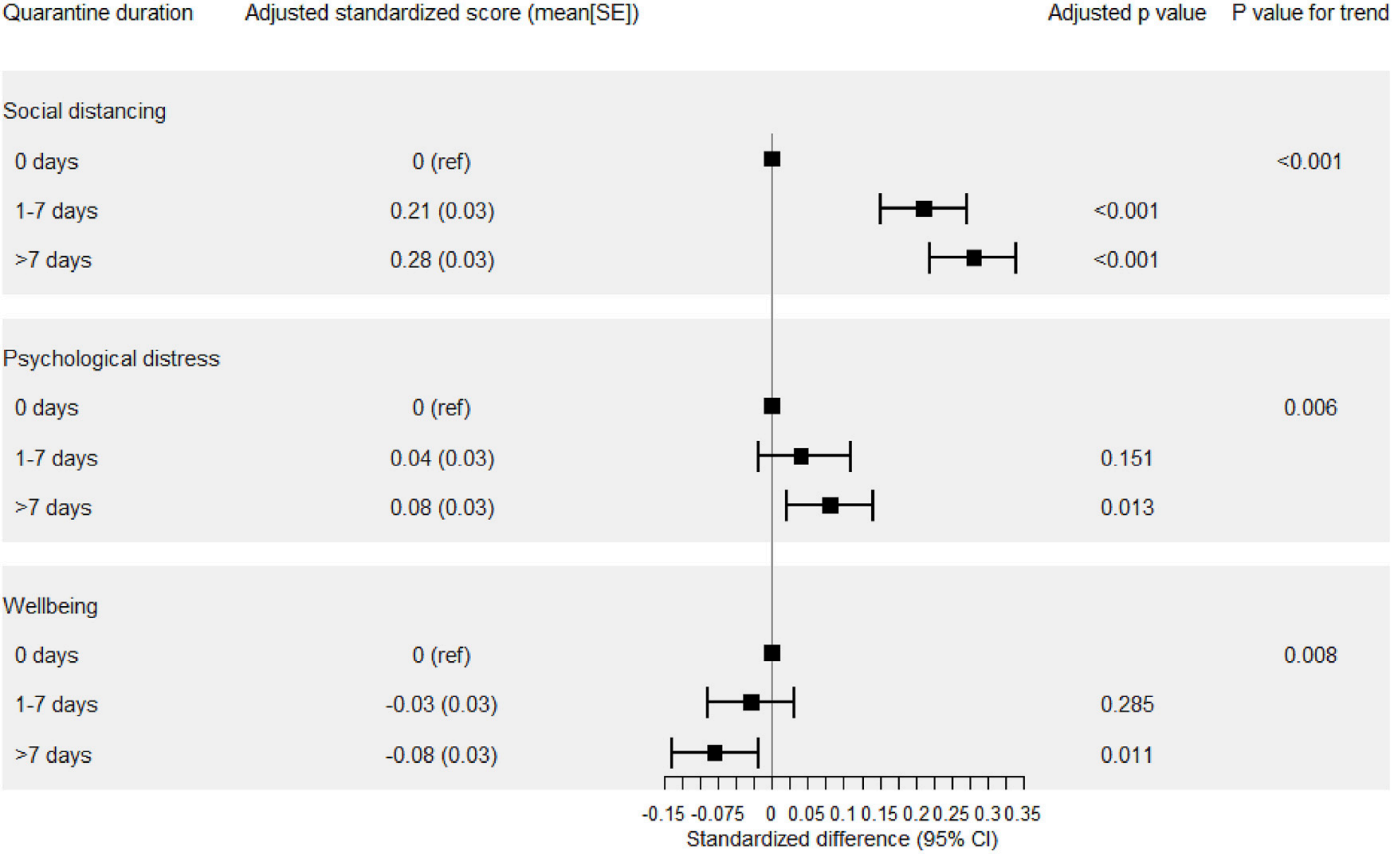
The adjusted standardized score of social distancing, psychological distress, and wellbeing was derived from linear regression, adjusted for gender, age, residence, marriage, education, income, health status, and chronic diseases (Collected from 10 to 23 January 2021, China).

The multiple linear regression (Table 3) shows that quarantine for 1–7 days increased the social distancing by 2.61 (95% CI = 1.90–3.33), whereas quarantine for >7 days increased the social distancing and psychological distress by 3.00 (95% CI = 2.37–3.64) and 1.03 (95% CI = 0.22–1.86), respectively. However, quarantine for >7 days decreased wellbeing by 1.27 (95% CI = 0.29–2.26). Figure 1 shows the standardized differences in social distancing, psychological distress, and wellbeing. Quarantine duration shows a dose-response relationship (*p* trend <0.01), indicating that individuals with longer quarantine duration reported increased social distancing, increased psychological distress, and decreased wellbeing during the COVID-19 pandemic. The multiple logistic regression results showed that the participants, who had been quarantined for 1–7 days, were 2.16 times more favorable to take the vaccine than those without quarantine after adjusting for parameters such as gender, age, residence, marriage, education, income, health status, and chronic diseases (Table 3).

**TABLE 3:** Multivariate analyses of association between quarantine duration and psychological distress, wellbeing social distancing as well as vaccination intention (Collected from 10 to 23 January 2021, China).

Quarantine duration (days)	Psychological distress		Wellbeing		Social distancing		Vaccination intention	
	*β* (95% CI)^a^	*β* (95% CI)^b^	*β* (95% CI)^a^	Β (95% CI)^b^	*β* (95% CI)^a^	*β* (95% CI) ^b^	OR (95% CI)^a^	OR (95% CI)^b^
0	Reference	Reference	Reference	Reference	Reference	Reference	Reference	Reference
1–7	0.64 (−0.32–1.59)	0.68 (−0.25–1.60)	−0.55 (−1.72–0.61)	−0.61 (−1.72–0.51)	2.56 (1.83–3.28)***	2.61 (1.90–3.33)***	1.91 (1.10–3.33)*	2.16 (1.22–3.82)**
>7	1.41 (0.58–2.25)**	1.03 (0.22–1.86)*	−1.46 (−2.48–0.44)**	−1.27 (−2.26–0.29)*	3.17 (2.53–3.80)***	3.00 (2.37–3.64)***	1.84 (1.14–2.97)*	1.58 (0.96–2.60)

a Unadjusted model.

b Adjusted for gender, age, residence, marriage, education, income, health status, and chronic diseases.

*p < 0.05; **p < 0.01; ***p < 0.001.

## Discussion

The general effects of quarantine have been discussed in previous literatures [12, 13, 23]. However, the effects of quarantine duration on mental health, social distancing, and vaccination intention were less discussed, especially during the second outbreak of COVID-19 in China. The present cross-sectional study showed that people suffered from mental health problems, especially those who were quarantined for more than 7 days, during the second outbreak. Furthermore, quarantine duration showed a dose response relationship with social distancing, psychological distress, and wellbeing. The participants who had been quarantined for 1–7 days had high intentions take a COVID-19 vaccine.

Furthermore, we found that the longer quarantine duration was associated with higher psychological distress and lower wellbeing, supporting a Durkheimian approach that the disruption of social networks has deleterious consequences on mental health [24]. These findings explained the phenomenon of the high prevalence of psychological disorders reported during the COVID-19 pandemic [11, 21, 25]. For example, during the pandemic, the prevalence of anxiety and depression in the United Kingdom was 24.4% and 31.4%, respectively [25]. Moreover, the level of anxiety and depression had gradually increased during the lockdown in the participants with pre-existing diagnoses of mental disorders [25]. Importantly, participants who had been quarantined for >7 days but not 1–7 days showed a significant increase in psychological distress and decrease in wellbeing, which extended the observation from previous studies that mandatory quarantined status (binary measurement) increased the risk of psychological distress [12, 26]. Thus, optimizing quarantine duration and providing psychological assistance at appropriate times may help in reducing the level of psychological distress when implementing quarantine strategies.

We found the dose-response relationship between quarantine duration and social distancing in the current study. This result demonstrated that those who experienced quarantine did not show quarantine fatigue during the second outbreak which was consistent with the results of previous studies performed during the initial outbreak of COVID-19 [27]. This revealed that quarantine may be an effective measure in containing the spread of COVID-19. In addition, the standardized differences in social distancing (Figure 1) were larger than psychological distress or wellbeing. This result indicated that the decrease in social contact may be one of the most important and direct effects of quarantine strategies.

Finally, the vaccination intention in the present study (62.1%) was lower than that reported in previous studies before the availability of COVID-19 vaccines [15, 18]. The scarcity principle may explain this observation [28, 29]. COVID-19 vaccines are one type of scarce medical resource [30]; the scarcity of vaccines may increase the demand from people during the COVID-19 pandemic, especially at the onset of the outbreak and during the unavailability of COVID-19 vaccines. When the vaccines were available in China on 31 December 2020, and were free of charge to all citizens, the demand for them was low. This finding was consistent with a previous study performed in Hongkong [31]. In addition, the participants who had been quarantined for 1–7 days showed the highest vaccination intention. Thus, the administration of COVID-19 at the start of quarantine vaccines may reduce the psychological distress resulting from the longer quarantine duration.

Some limitations of the present study are discussed while interpreting our findings. First, the cross-sectional design limits our ability to draw a causal conclusion. Longitudinal data analysis may better explain the relationship between quarantine and physiological distress as well as vaccination intention. Second, the use of an online survey in the present study may limit the sample representativeness. However, the online survey was more feasible and flexible than an offline survey during the COVID-19 pandemic. Moreover, an online survey has been conducted in previous studies [11, 18]. Third, some other variables such as loneliness may account for the relationship observed in this study. Previous studies showed that loneliness may predict mental symptoms during the COVID-19 pandemic [32, 33]. A wider range of variables should be considered to avoid potential confounding in future studies. Finally, self-reported data were used in the study; hence, memory and recall biases may exist.

### Conclusion

To conclude, this study explored the effects of the different lengths of quarantine on psychological outcomes and vaccination intention during the second outbreak of COVID-19 in China. Quarantine with a longer duration (>7 days) was associated with the increase in psychological distress and the decrease in wellbeing, whereas quarantine for 1–7 days was associated with high vaccination intention. Though quarantine is deemed necessary to limit COVID-19 spread, the duration of quarantine should be considered by policy-makers when strategizing immunization programs against COVID-19 and preventing psychological disorders during the COVID-19 pandemic.
